# A short review on the potential of coffee husk gasification for sustainable energy in Uganda

**DOI:** 10.12688/f1000research.10969.1

**Published:** 2017-10-06

**Authors:** Gilbert John Miito, Noble Banadda

**Affiliations:** 1 Department of Agricultural & Bio-systems Engineering, Makerere University, Kampala, Uganda

**Keywords:** Agricultural Biomass, Coffee Husks, Gasification, Wood Fuel, Energy Recovery

## Abstract

Agricultural biomass is widely recognized as a clean and renewable energy source, with increasing potential to replace conventional fossil fuels in the energy market. Uganda, like other developing countries, has a high dependency (91%) on wood fuel, leading to environmental degradation. With a coffee production of 233 Metric Tonnes per annum, relating to 46.6 Mega Tonnes of coffee husks from processing, transforming these husks into syngas through gasification can contribute to resolving the existing energy challenges. The objective of this article is to briefly review the energy potential of coffee husks through gasification, and how the gasification process could increase energy recoveries for coffee farmers. Previous  findings indicate that the 46.6 Mega Tonnes per year of coffee husks generated in Uganda, with a heating value of 18.34 MJ/kg, is capable of generating 24 GWh of energy. This will address a 0.7% portion of the energy situation in Uganda, while protecting the environment.

## Introduction

### Energy demand in an African context

Energy demand is rising rapidly, mainly due to population increase and increasing industrial activities. This demand has led to an increase in prices of major energy sources, for instance fossil fuels ^1– 4^. Use of fossil fuels causes air pollution, which, over time, escalates climate change effects as emissions of greenhouse gases and other pollutants increases ^5– 8^. Due to the increase in energy demand and prices of fossil fuels, researchers are motivated to discover additional viable energy sources. A case of interest is Fernandes and Costa ^9^, who noted that biomass, including wood agro residue, in Portugal has an annual energy potential of 160 Tera joules (TJ). In Mozambique, Vasco and Costa ^10^ pointed out that agricultural biomass can cater for 32% of the country’s energy demand.

Many sub-Saharan African (SSA) countries use agricultural biomass to cover most of their energy needs ^5^, but the efficincies are low, and thus the raw material usage is high ^11^. In Uganda, for example, woody biomass is used as wood fuel to provide for over 93% of the country’s energy needs ^12^. This reliance on wood fuel significantly increases the rate at which the country’s forest cover is shrinking, and is associated with the adverse change in weather patterns and consequent climate variability experienced in the country ^13^. With Uganda’s population growing at a rate of 3.2% per annum ^14^, the pressure on the country’s forests is bound to increase if the country’s dependence on wood fuel continues. On the other hand, alternative biomass sources, like agricultural and saw mill waste, and technologies, like pyrolysis and gasification, remain unexplored. Coffee, which is Uganda’s top-earning export crop, is widely grown throughout the country, and yielded 233 mega tons (MT) in 2014 ^15^. During the processing of the coffee, a substantial bulk of coffee husks are generated. In total, 46.6 MT of coffee husks were generated from the 233 MT of coffee produced, based on a 0.2 waste factor for coffee ^16^. These husks are currently being used as beddings in poultry units, farmers also use coffee husks to replenish nutrients in pineapple and banana gardens across the country and to a smaller extent for use in briquetting. However, the current uses amount to underutilization of the coffee husks in monetary and energy terms, and there is thus a need to improve coffee waste handling alternatives that encompass maximum utilization of the energy component of the waste, while also addressing the challenges associated with the use of woody biomass for energy reliance. This won’t only address the energy crisis in the country, but also increase the economic value of the husks ^17^.

Direct use of coffee husks as an energy source is hindered by the low efficiency of the energy recovery systems used. It is essential to transform the husks to a form that improves energy recovery. One process that can be considered is gasification, which involves alteration of compact carbonaceous fuel, in this case being the husks, into ignitable gas by partial incineration at elevated temperatures and moderate heating rates ^11, 18^. Through this process, coffee husks can be thermally converted to producer gas. Producer gas is a blend of carbon monoxide, hydrogen, methane, carbon dioxide and nitrogen. Producer gas is multipurpose in its use, as opposed to the solid biomass, from which it is derived ^19^. This article reviews the potential for coffee husks, as an alternative to wood as feedstock, for gas fuel production through gasification in Uganda.

## The energy situation in Uganda

### Electricity grid

According to Karekezi and Kithyoma ^20^, Africa is the least electrified continent in the world, and, in East Africa, Uganda is amongst the least electrified countries ( Table 1). Uganda presently has one of the lowest electricity consumption per capita in the world at 215 kWh per annum ^21^. The typical value for SSA is 552 kWh per capita, while that for the world is 2,975 kWh per capita ^22^. This low electricity coverage is attributed to electric tariffs and the fact that the electricity grid is concentrated in urban areas. Hence, people have continued to use wood fuel for their energy needs.

**Table 1.** Electrification patterns and Energy consumption in East African countries ^31^.

Country	Energy consumption (% households)	Electrification (% households)
	Biomass	Other
Kenya	70	30
Rwanda	90	10
Tanzania	90	10
Uganda	93	7

**Figure 1.**
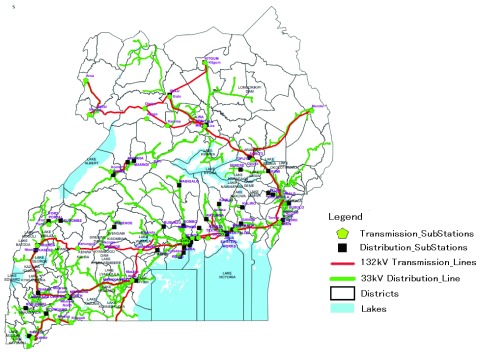
Uganda electric grid and sub stations. Adapted from 32.

An examination of the power grid in East Africa indicates that the spread of electricity is mainly limited to main town areas, while rural regions have no access ^23^ ( Figure 1). In addition, some homesteads that are close to the network lines cannot afford electricity connections and user fees. Indeed access to electricity at a national level in Uganda is about 15%, which is lower than other SSA ^24^, with a rural coverage of 1% ^5^. As a result, many Ugandans depend on firewood and charcoal as their main energy source for lighting and cooking ^25– 27^. According to the Rural Electrification Agency, the total woody biomass consumption in Uganda in 2013 was 31.7 million tons. The total energy consumption per year in Uganda is approximately 45.1 million tonnes of wood, 1.2 million m ^3^ of oil products, with a hydropower installed capacity of about 691.5 MW and 100 MW of thermal power ^28^.

### Fossil fuel

**Figure 2.**
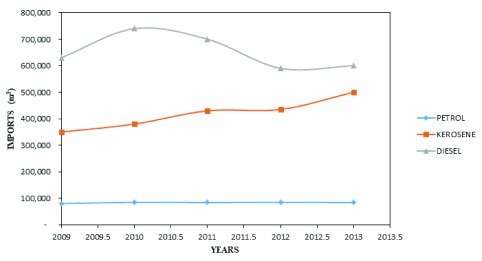
Petroleum product importation in Uganda ^15^.

A small portion (7%) of the energy demand in Uganda is met by fossil fuel ^29^, which is primarily used for automobiles and generators, and, to a smaller extent, in form of natural gas. Although fossil fuels contribute a small portion of the energy demand, data shows that there is increase in the volume of petroleum products imported into the country. In 2013, there was an increase of 4.6 and 4.3% in the import volume of kerosene and diesel, respectively ( Figure 2) ^15^. This increment implies an increased reliance on an energy source that is unsustainable. From Figure 2, it is also observable that there was a greater increase in the amounts of kerosene imported compared with other energy sources. This is highly attributed to the higher demand for kerosene, which the majority of the rural population uses for lighting ^30^.

### Biomass energy

**Figure 3.**
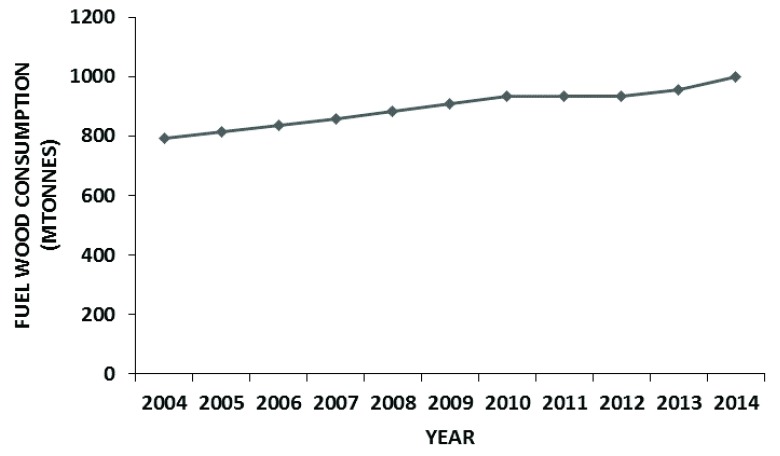
Trend of fuel wood consumption in Uganda ^33^.

Uganda is an energy deprived nation with restricted access to electricity and heavy dependence on wood fuel to cater for the energy requirements. There has been a general increase in the wood consumption for fuel in the country ( Figure 3) ^33^. This heavy consumption of wood fuel has contributed to high rates of deforestation, further leading to unreliable rainfall and rampant soil erosion ^34^.

Charcoal is largely used in metropolitan areas, whereas firewood, agricultural biomass and wood chippings are commonly utilized in rural areas ^35^. The sugar industry is the sole industry in Uganda that exploits agricultural biomass in the form of bagasse for cogeneration, but the technology should be adopted even for energy production in homesteads.

### Renewable energy

The promotion of renewable energy has been recognized as a potential method of addressing power shortages in the East African region ^20^. The renewable energy policy of Uganda states that the increasing cost of fossil-based fuels makes them costly for developing countries, and fossil fuels have an unreliable future. Uganda has extensive renewable energy resources for production of energy and the delivery of energy services, such as biomass, geothermal, large scale hydro, hydro, wind and solar energy, yet these remain un-tapped, primarily due to the apparent technical and financial incapacitations. With the exclusion of biomass, whose energy contribution is significant, the other sources contribute ~5% of the country’s total energy consumption. This hinders the scope and productivity of economic activities that can be undertaken in any part of the country. Biomass is an extremely available resource, and agricultural waste, such as coffee husks, is a large quota of the biomass yield. Therefore, it is advisable that the use of these readily abundant resources should be increased. 

### Status of gasification in Uganda

Gasification technology is not extensively used nor known in Uganda ^11^, but in Kenya, a neighboring country of Uganda, gasification of sawmill dust has been implemented with a power output of 76 GWh ^36^. Although previous investigations showed that small-scale wood gasifiers could be economically and socially feasible energy systems to generate electricity in rural areas ^37^, it is not a widely implemented technology in SSA. Obua *et al.* ^38^ noted that gasification is not widely applied in Uganda and reported only two established gasification units: one at Muziizi Tea Estate for electricity generation using wood feedstock and the other at Paramount Cheese Diaries for industrial heat production, which uses papyrus reeds. The gasification unit at Muziizi Tea Estate was stated to be 87 kW average power output, although rated at 200 kW ^39^, indicating a low operating efficiency. Buckholz *et al.* ^37^ described the economics of a 10 kW wood gasification unit in Mukono, Uganda, and concluded that the technology is proven, efficient and economically viable. The existing gasification units in Uganda rely on firewood, which is a factor in deforestation, and yet the literature supports use of coffee husks. Previous studies in Sweden ^40^ and Portugal ^41, 42^ have reported successful gasification of coffee husks for various applications, including fuel, electrical generation and soil conditioning, but the possibility of the technologies in less developed countries is still abstract.

## Deforestation situation

**Figure 4.**
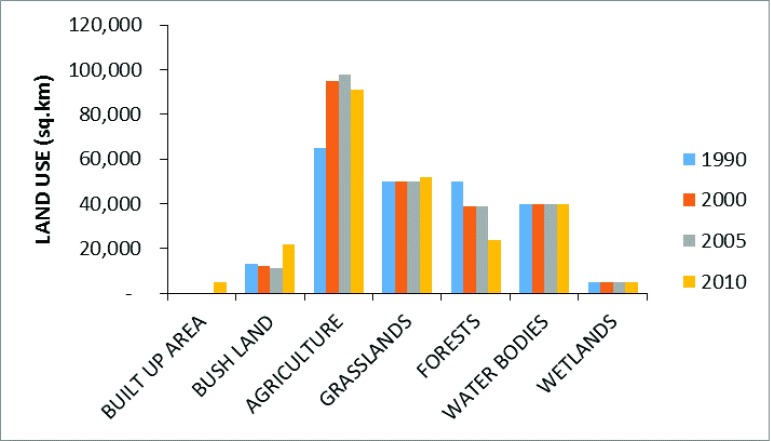
Area for land cover by type for the years 1990–2010 ^15^.

Uganda has an expanse of 241,550.7 square kilometers, including water bodies. Forests are one of the chief land uses although there has been a significant decline in forest area over the past two decades ^5, 43^. Between 1990 and 2010 total forest cover dropped from 24 to 12.7% of the total land area, implying an average decline of close to 2% per year ( Figure 4) ^28^. Although agriculture is the major cause of deforestation, wood extraction for energy production, which has been steadily rising over the years, has been noted as one of the direct reasons for deforestation ^38^. The forest area reduction contributes to environmental hazards and unreliable rainfall manifested in the country. Currently, approximately 90,000 ha of forest and vegetation are destroyed yearly, leading to fuel wood shortage in rural areas and increasing prices of charcoal and fuel wood ^15^.

## Coffee production in Uganda

**Figure 5.**
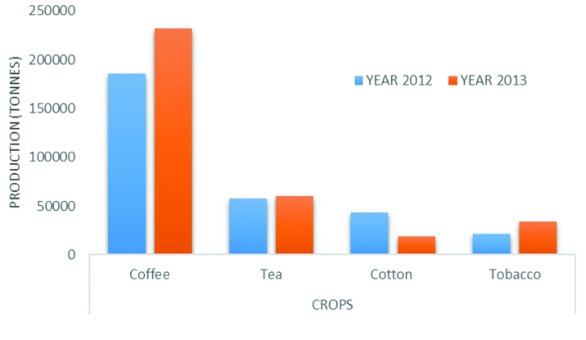
Cash crop production of Uganda ^15^.

**Figure 6.**
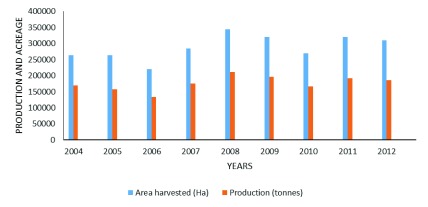
Coffee production in Uganda ^33^.

Uganda is ranked second to Ethiopia in regards to coffee production in Africa ^44^, and there is a current government intervention of coffee replanting, which is bound to boost coffee production in Uganda ^45^. Uganda produces two types of coffee: Robusta coffee and Arabica coffee (also termed as Mountain coffee). Over the years, Robusta coffee has been produced in larger quantities and accounts for 80% of production compared with Arabica coffee. In 2013, Uganda produced a sum of 232,561 tons of coffee ( Figure 5) from a producing area of about 310,000 hectares, of which 75% was Robusta ^15^. Currently, the ( Figure 5 and Figure 6) ^15^.

**Figure 7.**
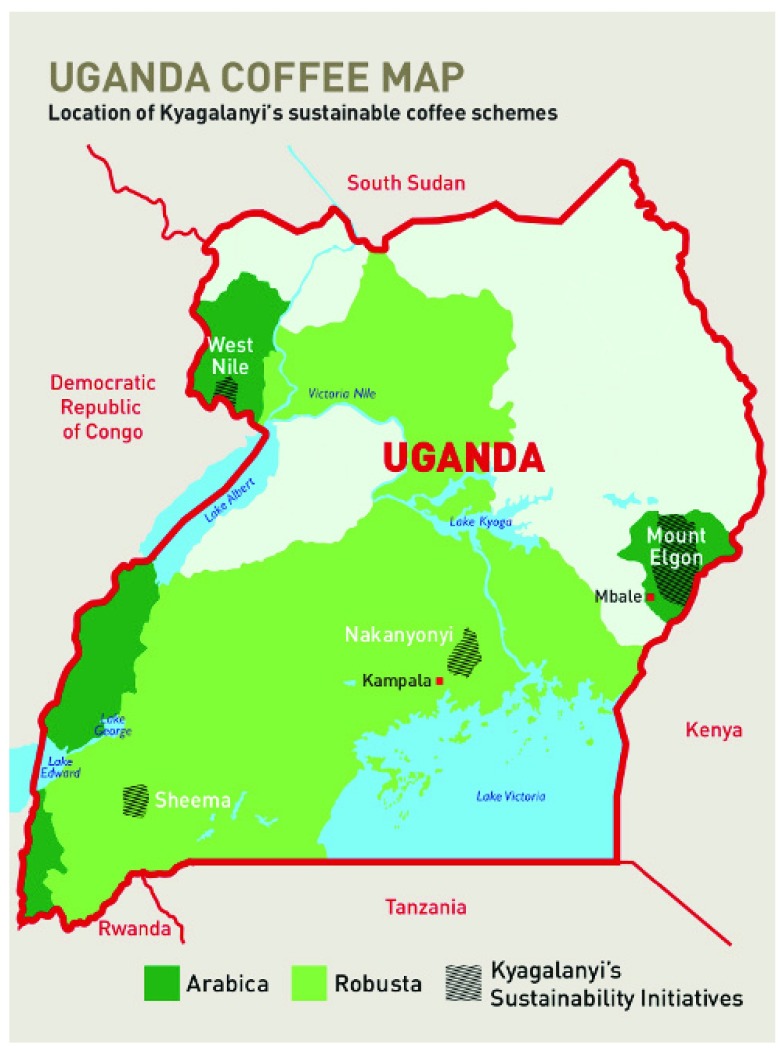
Coffee growing areas. Permission to use this figure was granted by Kyagalanyi Coffee Ltd ( http://kyagalanyi.co.ug/sustainability/sustainable-coffee-schemes/).

The major coffee growing regions include the Western Highlands, Bugisu around Mt. Elgon and areas around Lake Victoria basin ( Figure 7). According to Lora and Andrade ^16^, coffee has a waste factor of 0.2 by weight, and as such this yields a coffee husk production of approximately 46.6 MT per year ^46^. These husks are used as soil conditioners, briquetting, poultry bedding, and some are burnt as biomass energy sources, but with low efficiency. Significant to note is that the production surpasses the use of the husks ^24^.

## Gasification potential of coffee husks

According to Acharya *et al.* ^47^, processing of coffee yields about 22% of its weight as coffee husk. The annual generation of coffee husks in Uganda is estimated to be 46.6 M tons per annum, rising from 172 M tons per annum reported in 2004 ^48^. According to Mhilu ^40^, coffee has an energy value of 18.34 MJ/kg. At a maximum this would harness efficiencies of 855 TJ with 46.6 M tons of coffee husks. At a conversion efficiency of 65% ^46^, the generated coffee husks have a potential of 24 GWh energy production. This would translate to better efficiencies and environmental protection, yet reducing the reliance on wood fuel.

A study was carried out by Pereira *et al.* ^49^ concerning the development of gasification stoves. They found that the gasification process yields emissions to the environment, but the amounts are small compared with fossil fuels and direct ignition of the biomass. Through gasification, the coffee husks can be put to an environmentally sound use, as the emissions associated with open burning are reduced ^50^. The technology has been shown to be viable for a variety of feedstock, including waste from paper mills, mixed plastics, forest industry waste and agricultural residues ^28^. The coffee husk gasification technology has the potential to better livelihoods and contribute to local progress by providing electricity access to societies in rural Uganda ^30^.

## Conclusions

This short review critically focusses on the fact that gasification of coffee husks can address a portion the energy demand problem in Uganda, while enhancing cleaner production. The review of the literature shows that coffee husks have the potential for conversion to clean gas fuels for energy generation on an industrial scale through gasification. If the renewable energy policy of Uganda emphasizes the adoption of highly efficient renewable technologies, the heavy reliance of wood for fuel will be curbed. The Government of Uganda should effectively implement the coffee replanting scheme. This will increase the coffee yield, which in turn will increase the amounts of coffee husks produced. These coffee husks, if properly harnessed, can be a clean energy alternative. Uganda has a massive potential to produce energy from coffee husks, as coffee is a major cash crop from the country. If utilized in a viable manner, coffee husks could contribute to 24 GWh of energy, while decreasing deforestation and environmental degradation in the country, which are associated with the current energy sources. The technology adoption will further improve the livelihood of the residents, as well as lessen the pressure on wood fuel. However, the possibility of sustainable energy derivation from coffee husks is attached to the increasing production of coffee by allocating more unutilized land to coffee growing and also growing of improved and high yielding coffee varieties. Nevertheless, emphasis should be also put on food crops to avoid food insecurity.
