# Supplementary material for: A short review on the potential of coffee husk gasification for sustainable energy in Uganda
Source: F1000Res. 2017 Oct 6;6:1809. [Version 1] doi: 10.12688/f1000research.10969.1 (PMC5717475; doi:10.12688/f1000research.10969.1)
Supplement: Peer review history

# Peer review: Referee response for version 1

**DOI:** 10.5256/f1000research.11824.r26767

**Published:** 2017-12-04

**Authors:** Innocent Nhapi

**Affiliations:** 1 Department of Environmental Engineering, Chinhoyi University of Technology, Chinhoyi, Zimbabwe

Thank you for giving me this opportunity to review this article. The review is well written and focused and gives a good overview of the subject and possibility of utilising coffee husk gasification for sustainable energy in Uganda. I only made a few notable observations which are outlined below:

 There are some sections with bold and factual statements which needs to be backed by literature. An example is on page 2, paragraph 2, where uses of rice husks in Uganda are discussed. Another example is this sentence from page 4: **With the exclusion of biomass, whose energy contribution is significant, the other sources contribute ~5% of the country’s total energy consumption.** Some factual statements are referenced but the authors can go on and find some statistics on coverage - an example is page 4, second paragraph.

 This sentence on page 5 is not complete: **Currently, the (Figure 5 and Figure 6) ^15^**

 Make this sentence more clearer in meaning: **The annual generation of coffee husks in Uganda is estimated to be 46.6 M tons per annum, rising from 172 M tons per annum reported in 2004** - what is rising here?

 The energy needs to be solved are distributed around Uganda and so also is the cultivation of coffee. The authors need to consider the logistical and economic implications of their proposal and recommend how the gasification plants and energy produced will be distributed for the benefit of the rural peasants who are currently using firewood as source of fuel.

 .

 .

I have read this submission. I believe that I have an appropriate level of expertise to confirm that it is of an acceptable scientific standard.

# Peer review: Referee response for version 1

**DOI:** 10.5256/f1000research.11824.r27378

**Published:** 2017-11-02

**Authors:** Jamidu H.Y. Katima

**Affiliations:** 1 Department of Chemical and Mining Engineering, University of Dar es Salaam, Dar es Salaam, Tanzania

The review is relevant considering the current energy situation i.e. dwindling of fossil fuel resources and the impact of the same to climate change. Use of sustainable renewable resource which are considered to be carbon neutral is very important.

 However, the current generation of coffee husks (only meeting 0.7% of energy demand) does not provide comfort for sustainable energy supply.  Although the paper discusses the intention of Uganda to increase acreage of coffee plantation, it does not discuss to what extent this will be done to meet even 20% of Uganda’s energy supply.

 Specific Comments

 Abstract

1. Line 4, the Authors are stating that Uganda is producing 233 Metric Tonnes of coffee, which produce 46.6 Mega Tonnes. These numbers do not make sense. Unless 233 are in Mega Tonnes
2. Line 5, insert “per year” between “husks” and “from”
3. Line 13, 0.7% of total energy from coffee husks do not tally with the use of “sustainable energy” – probably instead the Authors should use contribute to energy mix

 

 Introduction

1. Line 5, the Authors imply other pollutants contribute to greenhouse gases, they should be specific by mentioning which ones, otherwise they should change the statement
2. Second paragraph, second line, last word “efficiencies” is misspelled

 

 The Energy Situation in Uganda

 Fossil Fuels

1. Line 3, the Authors are making reference to use of Natural Gas. My understanding is that Uganda is not using NG, probably they wanted to mean “Liquefied Petroleum Gas (LPG)”

 

 Biomass Energy on page 4

1. Second paragraph, line 5, Authors should change “Should” to “could”

 

 Status of gasification in Uganda

1. Line 21 “ replace the word “electrical” with “electricity”

 

**Gasification potential of coffee husks** (page 5)

1. Line 2 the Authors states that the coffee yield 22% of its weight as coffee husks. When you consider that Uganda produces 233 Mega Tonnes, then the coffee husks do not compute to 46.6 Mega Tonnes
2. The statement ” The  annual  generation  of coffee husks in Uganda is estimated to be 46.6 M tons per annum, rising from 172 M tons per annum reported in 2004” does not seem correct. 172 Mega Tonnes is larger than 46.6 Mega Tonnes

Conclusion

1. Line 8, use of statement “the heavy reliance of wood for fuel will be curbed” is a heavy statement without qualification particularly when you consider that the coffee husks contribute only 0.7% of energy in Uganda
2. Line 12, insert the word “source” between “a” and “clean”

I have read this submission. I believe that I have an appropriate level of expertise to confirm that it is of an acceptable scientific standard.
